# Comparison of vaginal microbiota in gynecologic cancer patients pre‐ and post‐radiation therapy and healthy women

**DOI:** 10.1002/cam4.3027

**Published:** 2020-04-01

**Authors:** Despina Tsementzi, Angela Pena‐Gonzalez, Jinbing Bai, Yi‐Juan Hu, Pretesh Patel, Joseph Shelton, Mary Dolan, Jessica Arluck, Namita Khanna, Lesley Conrad, Isabelle Scott, Tony Y. Eng, Konstantinos T. Konstantinidis, Deborah W. Bruner

**Affiliations:** ^1^ Nell Hodgson Woodruff School of Nursing Emory University Atlanta GA USA; ^2^ School of Biological Sciences Georgia Institute of Technology Atlanta GA USA; ^3^ Department of Biostatistics and Bioinformatics Emory University Atlanta GA USA; ^4^ Radiation Oncology Winship Cancer Institute Emory University Atlanta GA USA; ^5^ Department of Obstetrics and Gynecology Emory University Atlanta GA USA; ^6^ Grady Memorial Hospital Atlanta GA USA; ^7^ School of Civil & Environmental Engineering Georgia Institute of Technology Atlanta GA USA

**Keywords:** 16S rRNA gene, gynecologic cancer, postmenopausal women, radiation therapy, vaginal microbiota

## Abstract

**Background:**

While the importance of commensal microbes in vaginal health is well appreciated, little is known about the effects of gynecological cancer (GynCa) and radiation therapy (RT) on the vaginal microbiome (VM) of postmenopausal women.

**Methods:**

We studied women with GynCa, pre‐ (N = 65) and post‐RT (N = 25) and a group of healthy controls (N = 67) by sequencing the V4 region of the 16S rRNA gene from vaginal swabs and compared the diversity and composition of VMs between the three groups accounting for potential confounding factors in multivariate analysis of variance.

**Results:**

Comparisons of cancer vs healthy groups revealed that *Lactobacillus* and *Bifidobacterium* have significantly higher relative abundance in the healthy group, while the cancer group was enriched in 16 phylogroups associated with bacterial vaginosis (BV) and inflammation, including *Sneathia*, *Prevotella*, *Peptoniphilus*, *Fusobacterium*, *Anaerococcus*, *Dialister*, *Moryella,* and *Peptostreptococcus*. In our sample, RT affected the α‐diversity and correlated with higher abundance of typically rare VM species, including several members of the *Lacnospiraceae* family, a taxon previously linked to vaginal dysbiosis. In addition to cancer and treatment modalities, age and vaginal pH were identified as significant parameters that structure the VM.

**Conclusions:**

This is among the first reports identifying VM changes among postmenopausal women with cancer. RT alone seems to affect several phylogroups (12 bacterial genera), while gynecological cancer and its treatment modalities are associated with even greater significant shifts in the vaginal microbiota including the enrichment of opportunistic bacterial pathogens, which warrants further attention.

## Graphical abstract

Vaginal microbial communities in postmenopausal women undergoing gynecologic cancer treatments have small but detectable differences compared to healthy controls, including higher diversity and abundance of opportunistic bacterial pathogens. Radiation therapy alone seems to affect several phylogroups (12 bacterial genera), while gynecological cancer and its treatment modalities (surgery and chemotherapy) are associated with even greater significant shifts in the vaginal microbiota including the enrichment of opportunistic bacterial pathogens, which warrants further attention.

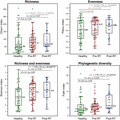

## 1 INTRODUCTION

The vaginal microbiome (VM) is a complex and dynamic ecosystem with a crucial role in the maintenance of a healthy vaginal microenvironment.^1^, ^2^ Reports from 16S rRNA gene (16S) amplicon sequencing studies of VMs from asymptomatic reproductive age women have described at least five community state types (CSTs), each of which is characterized by a specific composition and abundance of taxa. In contrast with other body sites, typical VMs and the corresponding CSTs exhibit low microbial diversity, typically dominated by one or few species of the *Lactobacillus* genus.^3^ Both host‐associated and environmental factors have been correlated with shifts in the composition of VMs, including estrogen levels, menstrual cycle, age, pregnancy, sexual behavior, hygiene, and even probiotic intake or diet.^4^, ^5^, ^6^ VMs in postmenopausal women are typically different from those of reproductive age women. Following menopause, estrogen levels drop, epithelial mucin production decreases and *Lactobacillus* colonization drastically decreases, resulting in increased vaginal pH,^5^, ^7^ These changes may render the female genitourinary tract more susceptible to infection and environmental disturbances.

Gynecologic cancers (GynCa) remain a major disease across the globe, with ~89 000 annual cases in the US alone, approximately 29 000 of which are terminal.^8^ The cost of therapies and the management of therapy‐related toxicities are major burdens.^9^ Microanatomical disruptions, gastric dysfunctions, mucosal atrophy, dyspareunia, and sexual dysfunction have been frequently reported for women undergoing treatment with radiation therapy (RT), and symptoms may persist even 2 years after the treatment.^9^, ^10^, ^11^ The disturbances of the VMs in women with gynecologic malignancies undergoing anticancer therapies remain understudied, due in part to the challenges associated with recruiting patients and obtaining appropriate samples. Both **GynCa** and **anticancer therapies** often have a major impact on vaginal pH and/or *Lactobacillus* colonization, and therefore, the health of the vaginal ecosystem.^12^, ^13^ Conversely, changes in the VMs may contribute to carcinogenesis, recurrence rates, or treatment‐related toxicities.^2^

In this study, we characterized VMs in postmenopausal women diagnosed with GynCa (cervical/endometrial) pre‐ and post‐RT and in healthy controls by 16S rRNA amplicon sequencing. We aimed to determine whether the VM structure and composition in women with GynCa is significantly different from that in healthy women, and if distinct community states discriminate vaginal microbiota pre‐ and post‐RT. We hypothesized that RT would be an additional factor contributing further to dissimilarities in the VMs.

## 2 METHODS

### 2.1 Study design

Informed consent was obtained from each participant and the study was approved by the Institutional Review Board. Clinical data were obtained from the medical records (Table S1). Eligibility included postmenopausal women (naturally or due to hysterectomy) with endometrial or cervical cancer treated with radiotherapy with or without surgery and/or chemotherapy, and without a history of other cancer or radiotherapy. Inclusion and exclusion criteria for cancer patients and healthy controls are provided in Table S2. Cancer patients received external beam radiation therapy (EBRT) delivered in daily fractions with total dose of 45‐50.4 Gy and/or intracavitary brachytherapy (IBT) delivered over 3‐5 fractions given twice weekly.

### 2.2 VM Samples

Vaginal swab samples were ascetically collected by a physician following standardized protocols from the Human Microbiome Project.^14^ Matched samples from each cancer patient were taken twice, first collected after cancer diagnosis, at least 4 weeks after surgery and prior to the start of RT (T0) and subsequently after the completion of RT (T1, 2‐4 months later). A distinct group of healthy controls was sampled only once during their annual clinical gynecological examination. Samples were obtained from the midvagina and stored in sterile MoBio Power Bead tubes (Mo Bio) at −80°C. DNA was extracted with the DNeasy PowerSoil Kit (Qiagen). PCR amplification of the V4 region was performed as described previously,^15^ and samples were sequenced using the MiSeq Reagent Kit v2 (Illumina). Two biological replicates from the first seven patients were sequenced for quality assurance.

### 2.3 16S rRNA gene amplicon analysis

The 16S rRNA gene sequences were processed as previously described to exclude adaptor reads, low quality, and chimeric sequences.^16^ Sample coverage was calculated using the Turing Good and Chao estimator (R package vegan).^17^ Reads were de novo clustered into operational taxonomic units (OTUs) at 97% nucleotide identity with UCLUST implemented in QIIME v1.8.0.^18^ Taxonomy was assigned with the RDP classifier, trained with the GreenGenes database (gg_13_8).^19^ The OTU table was normalized for sequencing depth using the cumulative sum scaling transformation (metagenomeSeq package).^20^

### 2.4 Diversity estimates and statistical analysis

α‐diversity was estimated using four complimentary metrics: Chao‐1 index to estimate OTU richness (number of total OTUs present in the sample), pielou index to estimate OTU evenness (similarity of abundances across OTUs), Shannon index (evenness and richness composite), and Faith's (PD) index to account for phylogenetic diversity.^17^ α‐diversity values were compared between GynCa and controls using the Kruskal‐Wallis test (independent samples) and between pre‐ and post‐RT samples using the Wilcoxon signed‐rank test (dependent samples).

β‐diversity analysis was performed using Bray‐Curtis dissimilarities (abundance weighted distance) and Jaccard distances (the presence/absence of detected OTUs‐not abundance weighted). Distance results were visualized through 2D NMDS ordination plots for visual inspection of similarities. Distance matrices were used to conduct permutational univariate and multivariate nonparametric analysis of dissimilarities (ADONIS2) using the R package *vegan* and *P*‐values were adjusted for multiple testing using the Bonferroni correction.^17^ Differentially abundant OTUs between groups of samples were identified using LEfSe with LDA score >3.0.^21^

### 2.5 Availability of data and materials

Raw sequencing files were deposited in NCBI (PRJNA448161). Clinical/demographic metadata are provided in the Table S1.

## 3 RESULTS

### 3.1 Study population

A total of 65 patients with GynCa (29 cervical, 36 endometrial) and 69 healthy postmenopausal women participated in this study (Table 1). VM samples were collected pre‐RT (T0, n = 65) and 1‐2 months post‐RT (T1, n = 25) from GynCa subjects, and only once from healthy women at time of routine gyn examination. GynCa and control groups had no significant differences in age, BMI, or racial distributions (Table 1). All subjects were postmenopausal and none were on hormone replacement therapy or antibiotic treatment during the course of the study.

**TABLE 1:** Clinical and demographic information of cancer patients and healthy controls

	n (%)				*P*‐value (cancer/controls)
	Cancer (n = 65)	Controls (n = 69)		Total (n = 134)	
Age in year, mean (SD)	56.1 (13.4)	59.3 (7.8)	57.9 (11)		.08 (unpaired *T* test)
Ancestry					
Caucasian	29 (44.6)	31 (44.9)	60 (44.8)		.51 (Fisher‐exact test)
African American	34 (52.3)	33 (47.8)	67 (50.0)		
Asian	2 (3.1)	5 (7.2)	7 (5.2)		
BMI (SD)	31.4 (7.6)	28.7 (7.9)	30.1 (7.8)		.02 (Kruskal‐Wallis)
Diagnosis					
Endometrial	36 (55.4)				
Cervical	29 (44.6)				
Type of treatment					
T0‐pre‐RT (n = 65)					
None	14 (21.5)				
Chemotherapy	9 (13.8)				
Surgery	24 (37.0)				
Surgery + Chemo	18 (27.7)				
T1‐post‐RT (n = 25)					
IBT + EBRT	9 (36)				
EBRT	4 (16)				
IBT	13 (52)				

Abbreviations: EBRT, external beam radiation therapy; IBT, intracavitary brachytherapy; SD, standard deviation.

### 3.2 High reproducibility of VM microbiome data

16S rRNA gene amplicon sequencing resulted in an average of ~25 000 reads per dataset. The estimated sample coverage, that is, the probability for a species of the community to be observed in the actual sequence dataset obtained, was nearly complete, with an average of 99.97% (Table S1). The methodological reproducibility was evaluated with replicated vaginal swabs, taken sequentially from the first seven patients. We found high agreement in community composition and abundance between each pair of replicates (average *R* ^2^ = .92, Figure S1) supporting the reliability of our data collection and analysis.

### 3.3 VM community structure in cancer and healthy samples

**FIGURE 1:**
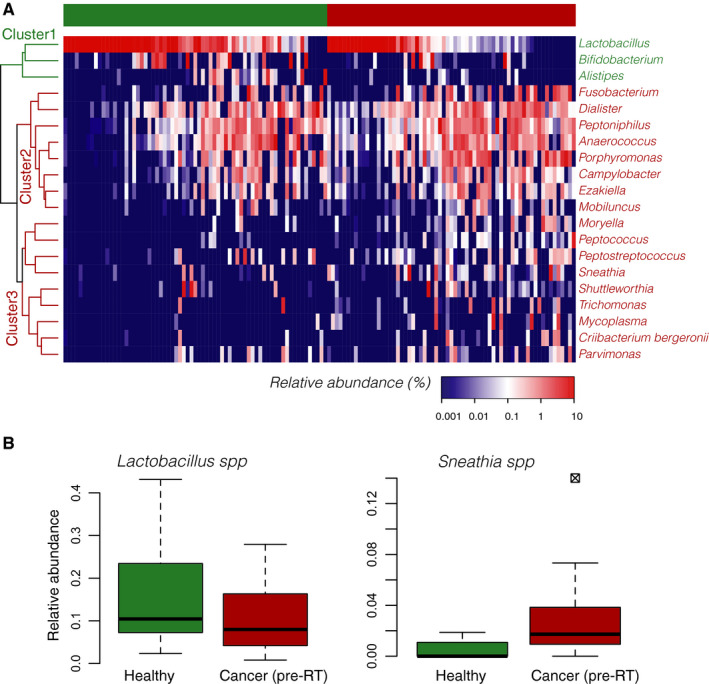
Vaginal microbiota in cancer (pre‐RT) and healthy groups. A, Heatmap showing hierarchical clustering of differentially abundant bacterial genera between the healthy and cancer group (n = 20). Three groups were observed: cluster I contained three genera significantly enriched in the healthy group, which included *Bifidobacterium*, *Allistipes,* and *Lactobacillus*. Cluster II grouped eight bacterial genera which were found in both groups but showed higher abundances in the cancer group overall. Finally, cluster III grouped nine genera which were observed mostly enriched in the GynCa group and completely absent from the majority of the healthy samples. B, Boxplots showing the estimated median relative abundance of *Lactobacillus* spp. in healthy and pre‐RT groups. Note that, in general, *Lactobacillus* spp. was more abundant in healthy than pre‐RT samples. C, Boxplots showing the estimated median relative abundance of *Sneathia* spp. in healthy and pre‐RT samples. A higher relative abundance of *Sneathia* spp. was observed in pre‐RT samples vs healthy. In panels (B) and (C), boxplots represent the first and third quartile and the horizontal segment represent the median value. RT, radiation therapy

Our study design included both dependent samples (individuals with cancer with some contributing samples pre and post‐RT) and independent samples (different individuals, ie, control vs cancer samples), thus we performed pair‐wise comparisons among three groups: healthy, pre‐RT, and post‐RT. A total of 521 OTUs were identified among all samples, the majority of which were categorized as rare community members (abundance <0.05%), and only 67 were found to have higher abundance in at least one sample. The low number of dominant OTUs observed reflected relatively simple VM communities in general, dominated mostly by *Firmicutes*, *Bacteroidetes*, and *Actinobacteria* (Figure S2). At the genus level, *Lactobacillus*, *Prevotella*, *Dialister*, and *Anaerococcus* were the most abundant members of the VMs (Figure 1A; Figure S3). As expected in postmenopause, only 15% of the VMs showed lactobacilli abundance >90% of the total community, while most VMs were comprised of multiple low abundance OTUs. Notably *Lactobacillus* was found to be predominant (>10% abundance) in 45% of the healthy VMs, but in only 32% of the pre‐RT GynCa samples (*P* = .04) (Figure 1B). *Sneathia*, on the other hand*,* was found identified as an abundant phylogroup (~5% rel. abundance) in the GynCa group and was absent or in very low abundance (<1%) in the healthy group (*P* = .001) (Figure 1C).

#### 3.3.1 Vaginal microbiota richness and diversity

**FIGURE 2:**
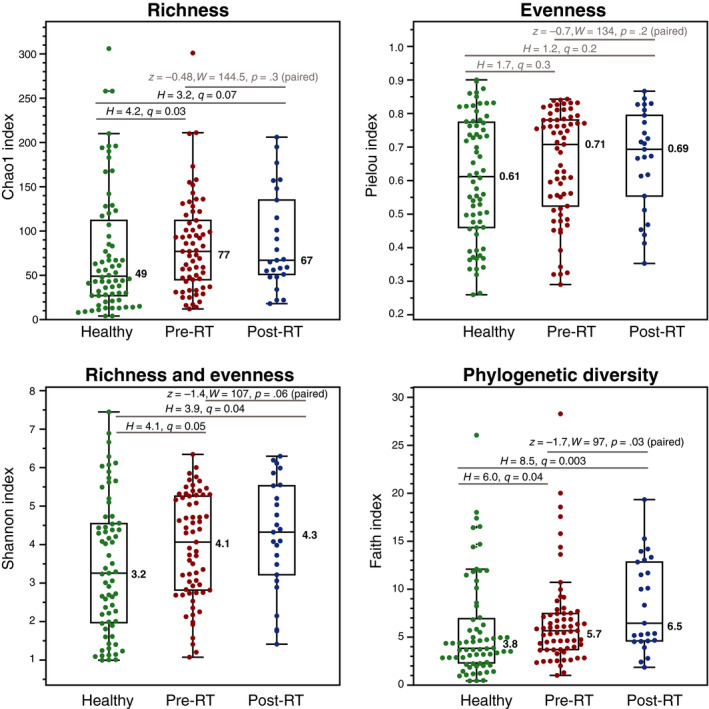
Comparison of community diversity metrics among healthy, pre‐RT and post‐RT cancer vaginal microbiome communities. A diversity overall increases from healthy to pre‐ and post‐RT cancer groups, a potential indication of disturbance in the microbial communities. Cancer samples show higher diversity than healthy samples in terms if richness, Shannon, and phylogenetic diversity. Post‐RT samples show slightly higher diversity than post‐RT samples in terms of Shannon and phylogenetic diversity. RT, radiation therapy

We estimated the α‐diversity of the VMs with four district metrics to quantify sample richness, evenness, and phylogenetic diversity (Figure 2). We observed higher α‐diversity in cancer with respect to healthy patients, and in post‐RT VMs with respect to pre‐RT. GynCa VMs had significantly larger number of OTUs (richness) compared to healthy VMs. Additionally Shannon index and phylogenetic diversity were increased in cancer patients (pre‐RT) compared to controls and were further increased in post‐RT VMs compared to pre‐RT. Thus, both GynCa and, to a lesser extent RT have a detectable, statistically significant effect on α‐diversity. No difference was observed in the evenness of the VMs from the different groups. Moreover, we did not identify any α‐diversity differences when comparing endometrial with cervical VMs (Figure S4), or when the comparisons were performed among Caucasian and African American VMs.

#### 3.3.2 Effects of clinical and demographic factors in VM composition

We performed univariate analysis of variance for each of clinical/demographic parameters on the β‐diversity distances within and between the three groups of samples (heathy, pre, and post‐RT cancer), to identify factors that might influence the VM composition (Table 2). Among all the parameters tested (Table 2; Table S1), we identified four parameters with a statistically significant marginal association on the community composition including cohort (healthy vs pre‐RT GynCa), subject, age, and pH. The marginal association of radiotherapy (pre vs post‐RT comparisons) was not significant at the microbial community level, a result corroborated by visual representation of VM differences in ordination analysis (Figure S5). The type of cancer treatment (surgery and/or chemotherapy, IBT, and/or EBRT), alcohol use, BMI, sexual intercourse within 4 weeks before sample collection, ancestry, and cancer stage or type (endometrial/cervical, Figure S4) had no detectable association with the variation among VMs. The results were consistent when comparing the VM variance using abundance‐based (Bray‐Curtis) or composition‐based distances (Jaccard). Overall, each of the significant parameters (healthy vs GynCa, age, pH) could only explain 1%‐2% of the observed variance among samples. On the other hand, 8% of the variance was found to be affected by the subject, highlighting the fact that intersubject variation is larger than the variation among groups.

**TABLE 2:** Univariate permutational analysis of variance (Adonis2, 100 K permutations) to quantify the marginal association of each parameter with the variability observed between vaginal microbiomes. Data for each parameter are provided in Table S1

Parameters	Bray‐Curtis (abundance‐based)			Jaccard (composition‐based)		
	N	*R* ^2^	*P*‐value	N	*R* ^2^	*P*‐value
Cohort	159	.012	**.003** ^a^	159	.010	**.004**
Subject	159	.882	**.001** ^a^	159	.868	**.001**
Radiotherapy	90	.013	.216	90	.007	.279
Age	159	.016	**.001** ^a^	159	.017	**.001**
Cancer type	90	.016	.082	90	.012	.086
Ancestry	159	.022	.104	159	.012	.081
BMI	158	.009	.061	158	.014	.059
pH	131	.028	**.001** ^a^	131	.021	**.001** ^a^
RT dose	153	.006	.727	153	.008	.558
Cancer stage	65	.012	.376	65	.020	.399
Smoking	111	.024	.221	111	.006	.429
Alcohol	103	.009	.687	103	.012	.399
Sexual intercourse (4W)	146	.010	.059	146	.021	.084
Pre‐RT treatment modality^b^	65	.012	.127	65	.008	.084

^a^ Significance level *P* < .01.

^b^ Treatment modality: surgery, surgery‐chemo, chemo, none.

Significant *P*‐values are bolded and underlined in the table.

To assess the relative contribution of demographic/clinical parameters that were previously identified as significant effectors of the VM composition, and in order to account for covariates, we conducted a multivariate nonparametric ANOVA of dissimilarities (ADONIS2) analysis with 100 000 permutations based on both Bray‐Curtis and Jaccard dissimilarity matrices. The results from the multivariate model (Table 3) corroborated the results from the individual univariate models, indicating that the effect of each parameter on the VM composition is not confounded by the others. We found that 2.8% of the variation observed among samples was explained by the vaginal pH and 1.7% by the subject's age. When controlling for those parameters, the cohort partitioning (healthy vs cancer) could explain another 1% of the observed variation. Finally, we repeated the multivariate analysis including each individual parameter (Table S1) after controlling for pH, age, and cohort, but we found no significant effect in the VM compositions. The remaining 90% of the VMs variation was apparently determined by different covariates not assessed by our data and measurements, and (high) interperson heterogeneity.

**TABLE 3:** Multivariate permutational analysis of variance (Adonis2, 100 K permutations) to quantify the combined effect of selected parameters in the variability observed between the vaginal microbiomes

Feature	Bray‐Curtis (abundance‐based)		Jaccard (composition‐based)	
	*R* ^2^ (%)	Pr (>*F*)	*R* ^2^ (%)	Pr (>*F*)
pH	.028	0.00001*	.025	0.00001*
Age	.017	0.0003*	.018	0.0002*
Cohort (healthy/cancer)	.011	0.02297*	.011	0.0213*
pH:cohort	.009	0.10	.008	0.23
Age:cohort	.007	0.41	.007	0.38
Residuals	.924		.931	
Total	1.000		1.000	

* Significance level *P* < .05.

### 3.4 Detection of differentially abundant taxa

**FIGURE 3:**
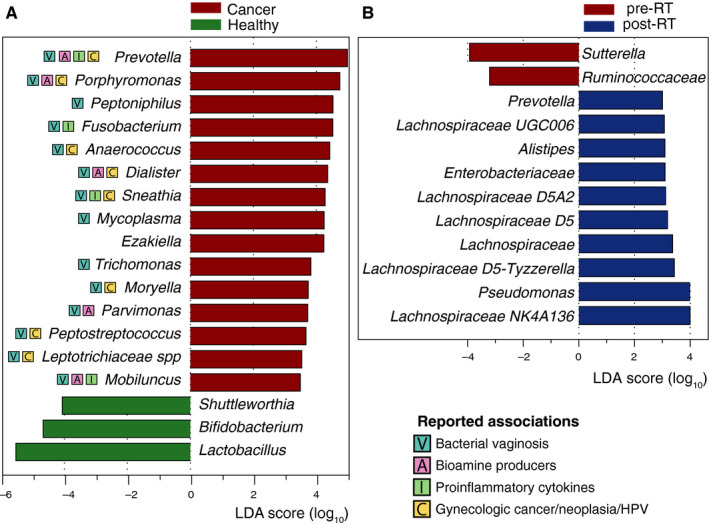
Differentially abundant bacterial operational taxonomic units between (A) healthy and cancer and (B) pre‐ and post‐RT vaginal microbiomes. Previously reported associations for each phylogroup are marked with squared indications and described in the discussion section. RT, radiation therapy

**FIGURE 4:**
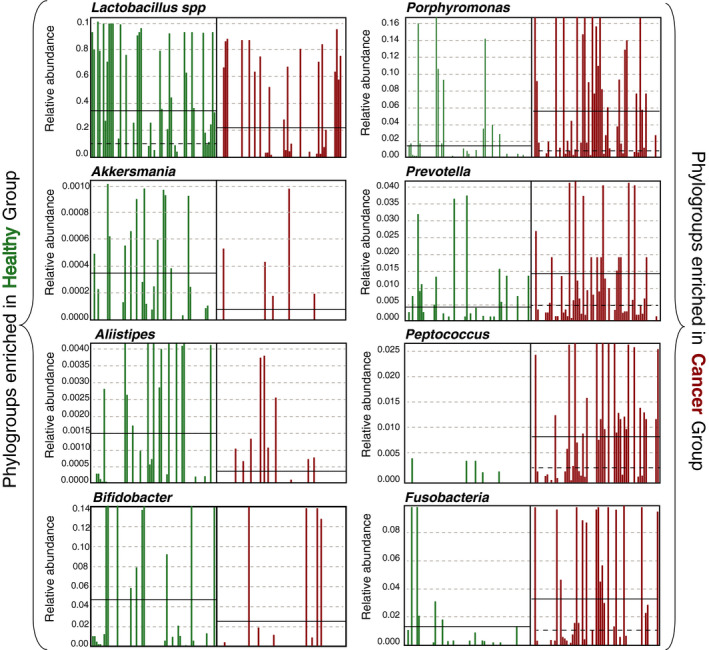
Detection of discriminative phylogroups in healthy vs cancer cohort. The plot compares the estimated relative abundance of eight selected operational taxonomic units discriminative of healthy (*Lactobacillus*, *Allistipes*, *Akkersmania,* and *Bifidobacter*) or GynCa groups (*Prevotella*, *Porhyromonas*, *Fusobacteria,* and *Peptococcus*). Continuous black lines correspond to the estimated mean relative abundances while striped black lines depict the median

While most of the parameters tested above did not seem to have a major impact on the β‐diversity variations, each parameter might affect specific bacterial taxa and not necessarily the total community structure. A biomarker discovery algorithm based on linear discriminant analysis (LDA) was employed (LEfSe) in order to identify discriminatory OTUs among the groups. LEfSe revealed 18 discriminative OTUs between healthy and pre‐RT GynCa groups (Figure 3). Manual inspection of the abundance profiles for selected discriminatory OTUs revealed that the abundance patterns were consistent for the majority of samples (as opposed to few outliers) confirming that the LEfSe was robust (Figure 4). Comparison of pre and post‐RT samples identified 12 differentially abundant phylogroups (Figure 3). Finally no differential abundant OTUs were detected when comparing cervical (n = 29) and endometrial cancer (n = 36) samples.

## 4 DISCUSSION

In this study, we characterized the vaginal microbiota of women postmenopause diagnosed with GynCa, pre‐ and post‐RT, and a group of healthy control samples. Our results revealed that VMs of GynCa patients differ from healthy controls, exhibiting higher microbial diversity and reduction of *Lactobacillus*. GynCa VMs were enriched in 15 phylogroups (Figure 3) that have been previously associated with dysmicrobiosis of the vagina (including BV, inflammation, cervical lesions, and/or endometrial cancer). While radiotherapy alone does not promote significant compositional shifts at the whole community level (β‐diversity), RT appeared to affect 12 individual phylogroups and consequently the α‐diversity; usually those phylogroups are low abundance VM members that are not typically encountered in a healthy VM environment. This is the first report in the literature of the detection of differentially abundant OTUs in cancer vs healthy; most notably, *Sneathia* was identified as a potential biomarker of postmenopausal women with cancer. Our findings are concordant with emerging literature on *Sneathia* as an opportunistic pathogen associated with dysbiosis and poor vaginal and reproductive outcomes in premenopausal women.^22^

### 4.1 Predictors of the vaginal microbiome structure

The age of the subject, vaginal pH, and partitioning into cancer vs healthy cohort was identified as the most important contributors that affect the VM structure (Table 2) independently without confounding effects (Table 3). All three parameters could only explain ~10% of the variability observed among VMs, and neither the cancer type, ancestry, treatment modality, nor BMI were found to have a significant effect on the VM at the community level. The remaining 90% of the variation remained unexplained and was apparently driven by covariates not assessed by our data and measurements, and/or high intrasubject variation. Our results suggest that the most significant determinant of the VM is the subject, indicating the differences between VMs among subjects (intrasubject variability) are significantly larger than the differences between groups. Those results are not surprising and have been previously described in various human microbiome projects. For example, metagenomic analysis of gut microbiomes from 1135 individuals indicated that a collection of 126 parameters (diet, antibiotics, lifestyle behaviors, clinical factors) can only explain 18.7% of the variation in microbial community compositions, while the rest of the variation is attributed to unaccounted factors and intrasubject variability.^23^

### 4.2 Microbial signatures of cancer

Cancer VMs showed higher α‐diversity (OTU richness and phylogenetic diversity) compared with controls (Figure 2), similarly to previous observations of increased diversity in VMs from cervical intraepithelial neoplasia or cancer (n = 149) compared to controls (n = 20).^24^ Increased α‐diversity of the VM is typically associated with pathogenic states, characterizing a community of multiple (high richness) low abundance (high evenness) species, as opposed to a balanced *Lactobacillus* dominated vaginal microbiome. Those results were corroborated by the multivariate analysis of variance, which identified that the cohort partitioning (healthy vs cancer) is a significant effector of the VM structure even when accounting for cofactors (Table 3). In other words, cancer vs healthy VMs have distinct community structures, and cancer VMs exhibit higher diversity, potentially a sign of VM perturbation.

Eighteen taxa accounted for most of the differences observed between healthy and cancer groups (Figure 3A). Among them *Shuttleworthia,* enriched in the healthy women, are typically encountered in nonlactobacillus‐dominated community state types,^3^ typically associated with BV states, but also commonly found in postmenopausal women.^5^, ^7^ *Bifidobacteria*, commonly found in healthy VM communities, are attributed a protective role similar to *lactobacilli*, that is, the production of lactic acid and hydrogen peroxide,^25^ preventing the overgrowth of pathogens and preserving the vaginal homeostasis. Depletion of *Lactobacilli* and *Bifidobacteria* in the cancer VMs suggests a suboptimal colonization which might indicate a state of dysmicrobiosis. Consistent with this hypothesis, 15 phylogroups commonly associated with a perturbed vaginal environment^1^, ^26^, ^27^ were more abundant in GynCa VMs. The majority of the phylogroups have been previously associated with bacterial vaginosis and/or production of bioamines,^28^ which can increase the vaginal pH and enhance the growth of other pathogens. Additionally, *Fusobacterium*, *Sneathia*, *Mobiluncus,* and *Prevotella* have been repeatedly correlated with the production of proinflammatory cytokines, with implications in cancinogenesis.^29^, ^30^ *Fusobacteria* and *Sneathia* typically inhabit mucous membranes and can invade epithelial cells, causing a wide range of human infections and eliciting host proinflammatory responses.^31^ Induction of proinflammatory cytokines might be directly correlated with cancer progression, and indeed several of the discriminatory phylogroups of the cancer cohort have been previously associated with GynCa or neoplasia (Figure 3A).^24^, ^30^, ^32^ Moreover, *Prevotella*, *Dialister*, *Sneathia,* and *Lachnospiraceae* have been correlated with persistence of HPV infections, which can lead to cervical cancer.^33^ *Sneathia* in particular, appears to be a distinguishing biomarker of GynCa according to our analysis; it exhibits the most pronounced differences between GynCa and controls and given the fact that it is an opportunistic pathogen of the human body it may play a significant role in vaginal and reproductive health.

### 4.3 Microbial signatures of RT treatment

Patients treated with radiotherapy for gynecologic malignancies often experience vaginal toxicity, including mucosal atrophy, and disruption in vaginal wall integrity.^9^ Not surprisingly, changes in the indigenous vaginal microbial community were also observed in our samples following RT (Figure 3B). The mechanism by which pelvic RT causes vaginal microbiota alterations remains unclear, but it might be related to changes in the relative abundance (or extinction) of key species producing mucopolysaccharides as glycosaminoglycans. We did not find significant differences in the overall community composition between pre‐ and post‐RT samples, but we did identify an increase in community richness and phylogenetic diversity as well as of the post‐RT appearance of low abundant species that are not typically found in VM community. Twelve phylogroups were found significantly enriched post‐RT, including six members of the *Lachnospiraceae* family. *Lachnospiraceae* have been associated with bacterial vaginosis, high‐risk sexual behaviors,^34^ and persistent genital tract inflammation.^35^ While *Prevotella* and *Pseudomonas*, enriched in post‐RT samples, are commonly encountered VM community members in postmenopausal women, the rest of the discriminatory phylogroups are typically rare members of the VM. The enrichment of rare community members observed in both pre‐ and post‐RT cancer samples might be an indication of further disturbance and a consequence of the depletion of *Lactobacillus*.

### 4.4 Limitations

This preliminary study had several limitations that restricted the generalizability of our findings despite being internally validated for quality assurance and potential contamination. The relatively small sample size analyzed here limited our ability to generalize our conclusions about all the different effects of radiotherapy on the VM, and how the recovery of the microbiome (if any) occurs over time. In addition, the low resolution of 16S rRNA marker gene did not allow us to identify phylogroups at the species or strain levels and distinguish pathogenic from commensal strains of the same species or genus. Finally, a larger cohort and possibly longitudinal sampling will be required to confirm these findings and identify the underlying mechanisms for the shifts in microbial diversity observed here.

### 4.5 Clinical significance

Our results are one of the first to reveal significant differences between healthy and GynCa VM states and identified discriminative OTUs that accounted for the observed differences. The functional consequences of these diversity shifts should be subject of future research. The perturbation of VM by RT associated with a decrease of *Lactobacillus* in the post‐RT group, are likely associated with some posttreatment symptoms, which has been previously observed in women with vulvovaginal atrophy. The results derived from this study, while preliminary, are among the first to assess changes conferred to the vaginal microbiome by gynecologic cancer and radiation therapy and could have implications for testing therapeutic interventions, such as probiotics or vaginal microbiome transplantation,^36^ that attempt to restore the ecology of the vaginal microbial community and/or help reduce patients’ suffering from treatment‐related symptoms.

## CONFLICT OF INTEREST

The authors declare no conflict of interest.

## AUTHOR CONTRIBUTIONS

Deborah W. Bruner was involved in conceptualization, funding acquisition, supervision, resources, and writing—review and editing. Konstantinos T. Konstantinidis was involved in conceptualization, funding acquisition, resources, and writing—review and editing. Despina Tsementzi was involved in formal analysis, data curation, methodology, visualization, and writing—original draft. Angela Pena‐Gonzalez was involved in formal analysis, and writing—original draft. Jinbing Bai, Yi‐Juan Hu, Lesley Conrad, Isabelle Scott, Pretesh Patel, Mary Dolan, Joseph Shelton, Jessica Arluck, Namita Khanna, and Tony Eng were involved in data curation and writing—review and editing.

## Supporting information

Fig S1‐S5

Table S1

Table S2
