# Supplementary material for: Comparison of vaginal microbiota in gynecologic cancer patients pre‐ and post‐radiation therapy and healthy women
Source: Cancer Med. 2020 Apr 1;9(11):3714–24. doi: 10.1002/cam4.3027 (PMC7286461; doi:10.1002/cam4.3027)
Supplement: Supplementary file 1 — Fig S1‐S5 [file CAM4-9-3714-s001.docx]

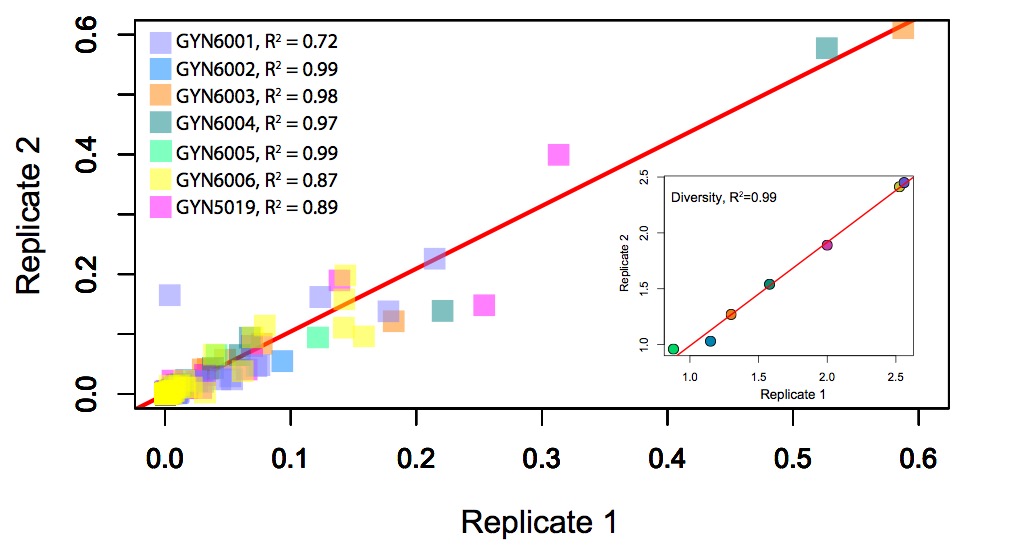


**Supporting Figure 1. VM correlation analysis between seven replicated samples**. The plot shows a linear model fitted to the estimated OTU abundance between replicates R1 and R2 for seven individuals. Individual OTUs are represented by data points, colored by the individual that they were recovered from, and the graph compares the relative abundance of each OTU in replicate 1 vs. replicate 2. The strength of the correlation is measured with the coefficient of determination R^2^. Inset plot shows the correlation analysis based on diversity estimates. In general, a high concordance in microbial community composition and diversity between replicated samples was observed.

**
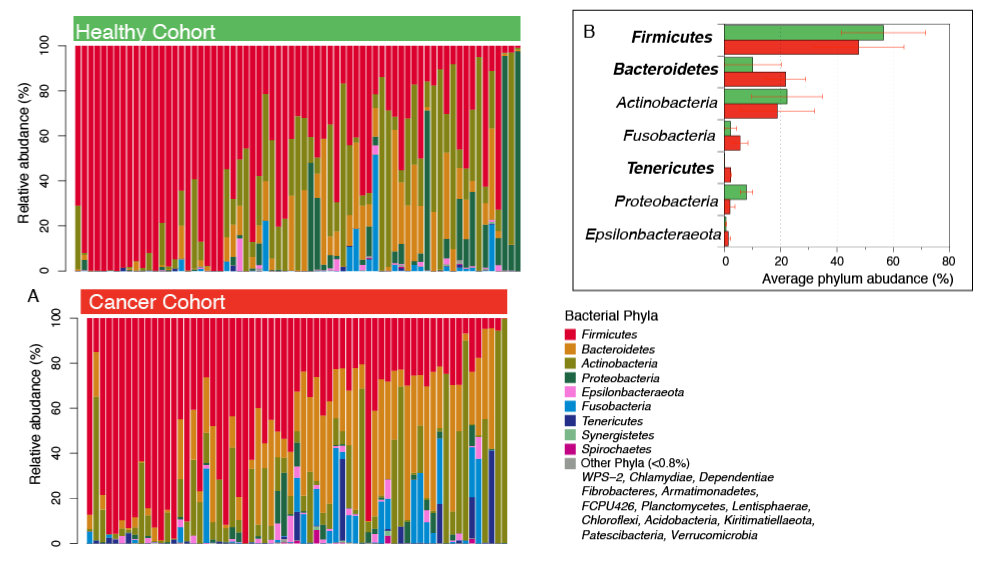
**

**Supporting Figure 2. Vaginal microbiota composition at the phylum level.** Barplots showing the vaginal microbiota composition and abundance at the phylum level in healthy (panel A) and GynCa individuals and the comparison of the average relative abundance per phyla (panel B). *Firmicutes* were found significantly more abundant in the group of healthy women than the pre-RT GynCa group (*p≤0.05,* Welch two sample t-test), while *Bacteroidetes* and *Proteobacteria* were more abundant in the pre-RT GynCa women (*Bacteroidetes*, *p=0.03*, *Proteobacteria*, *p=0.04,* Welch two sample t-test).

**Supporting Figure 3. Vaginal microbiota composition at the OTU level.** Barplots showing the vaginal microbiota composition and abundance at the OTU level (genus, 97% identity) in healthy (panel A) and GynCa post-menopausal women (panel B). Only the 20 most abundant phylogroups are shown.

**_
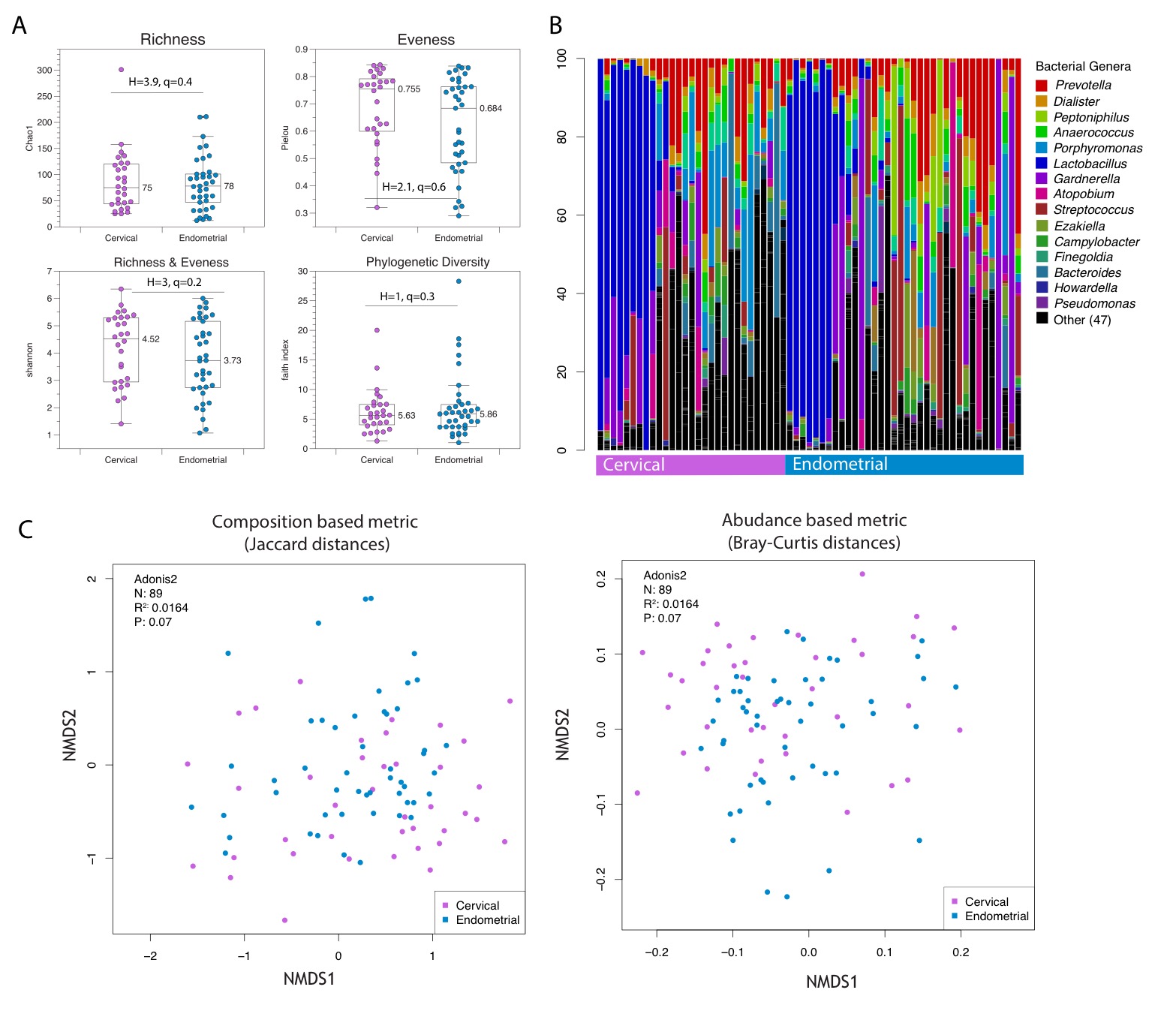
_ Supporting Figure 4. Community composition differences by cancer type. (A) Boxplots showing the estimated median values of evenness, richness, Shannon and phylogenetic diversity in cervical and endometrial cancer samples.** No significant differences were observed in community richness between both types of cancer (*Wilconson rank-sum test for independent samples*). (B) Relative abundance percent of dominating phylogroups at the genus level in cervical and endometrial cancer samples. (C) NMDS ordination plots based on Bray-Curtis and Jaccard distances.


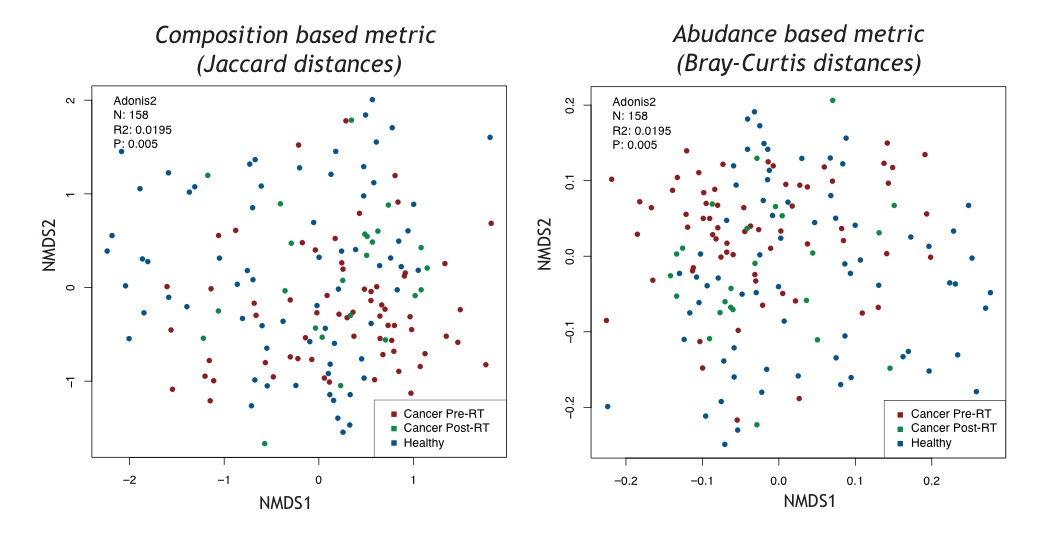


**Supporting Figure 5. NMDS ordination plots based on Bray-Curtis and Jaccard distances. Inter-sample variation analysis among healthy (blue), pre-RT (red) and post-RT (green) groups.** There is not a noticeable clustering among the groups, an indication that the intra-subject variation is larger than the variation among the groups.
