# Supplementary material for: Comparison of vaginal microbiota in gynecologic cancer patients pre‐ and post‐radiation therapy and healthy women
Source: Cancer Med. 2020 Apr 1;9(11):3714–24. doi: 10.1002/cam4.3027 (PMC7286461; doi:10.1002/cam4.3027)
Supplement: Supplementary file 2 — Table S1 [file CAM4-9-3714-s002.docx]

|  | | | | | | | | | | | | | | |
| --- | --- | --- | --- | --- | --- | --- | --- | --- | --- | --- | --- | --- | --- | --- |
| **Supportive Table 1**: Metadata and dataset quality metrics associated with each vaginal microbiome sample. | | | | | | | | | | | | | | |
| **Part A: Metadata** | | | | | | | | | | | | | | |
| **SampleID** | **Cohort** | **Subject** | **Time-point** | **Age** | **Canc. Type** | **Ancestry** | **BMI** | **PH** | **GY Dose** | **Canc. Stage** | **SMOKER** | **ALCOHOL** | **Sexual Intercourse 4W** | **Treatment** |
| gm001_2 | healthy | GEM1001 | T0 | 65 | NA | C | 20.0 | 6 | 0 | NA | NA | NA | NA | None |
| gm002_0 | healthy | GEM1002 | T0 | 73 | NA | C | 20.9 | 4 | 0 | NA | NEVER | 2 | Yes | None |
| gm003_0 | healthy | GEM1003 | T0 | 68 | NA | C | 39.7 | 6 | 0 | NA | NEVER | 1 | No | None |
| gm004_0 | healthy | GEM1004 | T0 | 71 | NA | C | 25.2 | 5 | 0 | NA | NEVER | 1 | Yes | None |
| gm006_0 | healthy | GEM1006 | T0 | 55 | NA | C | 26.3 | 7 | 0 | NA | PAST | 3 | No | None |
| gm007_0 | healthy | GEM1007 | T0 | 67 | NA | C | 27.6 | 4 | 0 | NA | PAST | 3 | Yes | None |
| gm008_0 | healthy | GEM1008 | T0 | 52 | NA | A | 23.8 | 4 | 0 | NA | NEVER | 1 | Yes | None |
| gm009_0 | healthy | GEM1009 | T0 | 61 | NA | AA | 23.3 | 6 | 0 | NA | PAST | 3 | No | None |
| gm010_0 | healthy | GEM1010 | T0 | 61 | NA | A | 25.7 | 6 | 0 | NA | NEVER | 1 | No | None |
| gm011_0 | healthy | GEM1011 | T0 | 62 | NA | A | 17.7 | 5 | 0 | NA | NEVER | 1 | No | None |
| gm013_0 | healthy | GEM1013 | T0 | 63 | NA | AA | 30.2 | 5 | 0 | NA | NEVER | NA | No | None |
| gm014_0 | healthy | GEM1014 | T0 | 72 | NA | C | 18.5 | 5 | 0 | NA | NEVER | 2 | NA | None |
| gm015_0 | healthy | GEM1015 | T0 | 55 | NA | C | 23.6 | 5 | 0 | NA | NEVER | 1 | Yes | None |
| gm016_0 | healthy | GEM1016 | T0 | 54 | NA | AA | 27.8 | 5 | 0 | NA | NA | NA | NA | None |
| gm017_1 | healthy | GEM1017 | T0 | 66 | NA | C | 24.2 | 5 | 0 | NA | NA | NA | Yes | None |
| gm018_0 | cancer | GEM1018 | T0 | 51 | C | C | 25.1 | 6 | 0 | 3 | CURRENT | 1 | No | Surgery + Chemo |
| gm018_1 | cancer | GEM1018 | T1 | 51 | C | C | 25.1 | 8 | 74 | 3 | NA | NA | Yes | iBT + EBRT |
| gm019_0 | healthy | GEM1019 | T0 | 58 | NA | C | 22.7 | 5 | 0 | NA | NEVER | 2 | Yes | None |
| gm020_0 | healthy | GEM1020 | T0 | 65 | NA | AA | 40.5 | 4 | 0 | NA | NEVER | NA | No | None |
| gm021_0 | healthy | GEM1021 | T0 | 58 | NA | C | 33.5 | 5 | 0 | NA | PAST | 2 | Yes | None |
| gm022_0 | cancer | GEM1022 | T0 | 68 | E | C | 17.5 | 8 | 0 | 2 | NEVER | 1 | No | Surgery + Chemo |
| gm022_1 | cancer | GEM1022 | T1 | 68 | E | C | 17.5 | 7 | 60 | 2 | NA | NA | No | iBT + EBRT |
| gm024_0 | cancer | GEM1024 | T0 | 64 | E | AA | 31.8 | 4 | 0 | 1 | NEVER | 1 | No | Surgery |
| gm024_1 | cancer | GEM1024 | T1 | 64 | E | AA | 31.8 | 5 | 22 | 1 | NA | NA | Yes | iBT |
| gm025_0 | healthy | GEM1025 | T0 | 64 | NA | C | 20.9 | 7 | 0 | NA | NEVER | 2 | No | None |
| gm027_0 | cancer | GEM1027 | T0 | 66 | E | C | 31.9 | 7 | 0 | 2 | CURRENT | 1 | No | Chemo |
| gm027_1 | cancer | GEM1027 | T1 | 66 | E | C | 31.9 | 7 | 127.5 | 2 | NA | NA | Yes | iBT + EBRT |
| gm028_0 | healthy | GEM1028 | T0 | 75 | NA | C | 25.8 | 7 | 0 | NA | NEVER | 3 | Yes | None |
| gm029_0 | cancer | GEM1029 | T0 | 45 | C | C | 25.5 | 5 | 0 | 2 | NEVER | NA | Yes | Surgery |
| gm030_0 | healthy | GEM1030 | T0 | 60 | NA | A | 34.9 | 5 | 0 | NA | NEVER | 1 | Yes | None |
| gm031_0 | healthy | GEM1031 | T0 | 71 | NA | C | 21.8 | 7 | 0 | NA | PAST | 1 | No | None |
| gm032_0 | healthy | GEM1032 | T0 | 72 | NA | C | 26.3 | NA | 0 | NA | PAST | 3 | NA | None |
| gm033_0 | healthy | GEM1033 | T0 | 63 | NA | AA | 24.5 | 4 | 0 | NA | PAST | 2 | Yes | None |
| gm035_0 | healthy | GEM1035 | T0 | 57 | NA | C | 36.9 | NA | 0 | NA | PAST | 2 | No | None |
| gm036_0 | healthy | GEM1036 | T0 | 66 | NA | AA | 27.3 | NA | 0 | NA | PAST | 1 | No | None |
| gm037_0 | cancer | GEM1037 | T0 | 54 | E | C | 28.8 | 5 | 0 | 3 | NEVER | 1 | Yes | Chemo |
| gm037_1 | cancer | GEM1037 | T1 | 54 | E | C | 28.8 | 5 | 45 | 3 | NA | NA | Yes | EBRT |
| gm038_0 | healthy | GEM1038 | T0 | 69 | NA | C | 32.1 | 5 | 0 | NA | NEVER | 1 | No | None |
| gm039_0 | healthy | GEM1039 | T0 | 70 | NA | C | 25.1 | 6 | 0 | NA | PAST | 1 | Yes | None |
| gm040_0 | healthy | GEM1040 | T0 | 58 | NA | AA | 40.4 | 6 | 0 | NA | NEVER | 1 | Yes | None |
| gm041_0 | cancer | GEM1041 | T0 | 30 | E | AA | 46.1 | 4 | 0 | 1 | PAST | 1 | Yes | None |
| gm042_0 | healthy | GEM1042 | T0 | 63 | NA | AA | 33.9 | 4 | 0 | NA | PAST | 1 | Yes | None |
| gm043_0 | healthy | GEM1043 | T0 | 56 | NA | C | 21.1 | 4 | 0 | NA | PAST | 1 | Yes | None |
| gm044_0 | cancer | GEM1044 | T0 | 74 | E | C | 34.5 | 7 | 0 | 3 | NEVER | NA | No | Surgery + Chemo |
| gm044_1 | cancer | GEM1044 | T1 | 74 | E | C | 34.5 | 5 | 45 | 3 | NA | NA | Yes | EBRT |
| gm045_0 | healthy | GEM1045 | T0 | 64 | NA | AA | 27.8 | 5 | 0 | NA | NEVER | 1 | Yes | None |
| gm046_0 | healthy | GEM1046 | T0 | 59 | NA | C | 29.3 | 5 | 0 | NA | NEVER | 1 | No | None |
| gm047_0 | healthy | GEM1047 | T0 | 67 | NA | C | 26.2 | 5 | 0 | NA | NEVER | 2 | No | None |
| gm049_0 | healthy | GEM1049 | T0 | 55 | NA | AA | 35.6 | 5 | 0 | NA | NEVER | 1 | Yes | None |
| gm050_0 | cancer | GEM1050 | T0 | 33 | E | AA | 40.8 | 5 | 0 | 1 | NEVER | 2 | Yes | Surgery |
| gm051_0 | healthy | GEM1051 | T0 | 69 | NA | C | 21.3 | 7 | 0 | NA | PAST | 2 | No | None |
| gm052_0 | healthy | GEM1052 | T0 | 51 | NA | C | 20.1 | 7 | 0 | NA | NEVER | 1 | Yes | None |
| gm053_0 | healthy | GEM1053 | T0 | 65 | NA | C | 19.7 | 8 | 0 | NA | NEVER | 3 | Yes | None |
| gm054_0 | cancer | GEM1054 | T0 | 69 | E | AA | 35.9 | 7 | 0 | 1 | NEVER | 1 | Yes | Surgery |
| gm054_1 | cancer | GEM1054 | T1 | 69 | E | AA | 35.9 | 6 | 25 | 1 | NA | NA | Yes | iBT |
| gm055_0 | healthy | GEM1055 | T0 | 58 | NA | AA | 26.4 | 5 | 0 | NA | PAST | 2 | No | None |
| gm056_0 | cancer | GEM1056 | T0 | 50 | C | AA | 30.9 | 4 | 0 | 1 | NEVER | 1 | No | Surgery |
| gm056_1 | cancer | GEM1056 | T1 | 50 | C | AA | 30.9 | 4 | 125 | 1 | NA | NA | Yes | iBT + EBRT |
| gm057_0 | cancer | GEM1057 | T0 | 40 | C | AA | 25.9 | 8 | 0 | 3 | CURRENT | 1 | No | Chemo |
| gm058_0 | cancer | GEM1058 | T0 | 37 | C | C | 23.7 | 5 | 0 | 2 | NEVER | 4 | No | Chemo |
| gm059_0 | cancer | GEM1059 | T0 | 39 | E | C | 37.6 | 5 | 0 | 2 | NA | NA | Yes | Surgery + Chemo |
| gm060_0 | healthy | GEM1060 | T0 | 69 | NA | AA | 18.4 | 8 | 0 | NA | NEVER | 1 | NA | None |
| gm061_0 | healthy | GEM1061 | T0 | 65 | NA | AA | 29.8 | 6 | 0 | NA | NEVER | NA | Yes | None |
| gm117_0 | healthy | GEM1062 | T0 | 67 | NA | AA | 42.8 | 5 | 0 | NA | NEVER | 1 | Yes | None |
| gm062_0 | healthy | GEM1062 | T0 | 67 | NA | AA | 29.8 | 4 | 0 | NA | NA | NA | Yes | None |
| gm063_0 | cancer | GEM1063 | T0 | 35 | C | AA | 28.1 | 8 | 0 | 1 | NEVER | 1 | No | None |
| gm064_0 | healthy | GEM1064 | T0 | 63 | NA | AA | 34.5 | 6 | 0 | NA | NEVER | 1 | No | None |
| gm065_0 | cancer | GEM1065 | T0 | 57 | C | C | 29.8 | 8 | 0 | 2 | NEVER | 1 | No | Chemo |
| gm065_1 | cancer | GEM1065 | T1 | 57 | C | C | 29.8 | 8 | 72.5 | 2 | NA | NA | No | iBT + EBRT |
| gm066_0 | cancer | GEM1066 | T0 | 65 | E | C | 33.8 | 5 | 0 | 2 | PAST | 1 | NA | Surgery + Chemo |
| gm067_0 | healthy | GEM1067 | T0 | 62 | NA | AA | 26.6 | 5 | 0 | NA | CURRENT | 1 | Yes | None |
| gm068_0 | healthy | GEM1068 | T0 | 46 | NA | AA | 28.5 | 5 | 0 | NA | NEVER | 1 | Yes | None |
| gm069_0 | healthy | GEM1069 | T0 | 50 | NA | AA | 48.1 | 5 | 0 | NA | NEVER | 2 | Yes | None |
| gm070_0 | cancer | GEM1070 | T0 | 34 | C | C | NA | 5 | 0 | 2 | PAST | 3 | Yes | None |
| gm071_0 | cancer | GEM1071 | T0 | 45 | C | AA | 46.5 | 4 | 0 | 2 | NEVER | 1 | No | Chemo |
| gm071_1 | cancer | GEM1071 | T1 | 45 | C | AA | 46.5 | 8 | 72.5 | 2 | NA | NA | No | iBT + EBRT |
| gm072_0 | healthy | GEM1072 | T0 | 60 | NA | AA | 47.6 | 5 | 0 | NA | NEVER | 3 | Yes | None |
| gm073_0 | cancer | GEM1073 | T0 | 68 | E | C | 47.6 | 7 | 0 | 3 | NA | NA | No | Chemo |
| gm073_1 | cancer | GEM1073 | T1 | 68 | E | C | 47.6 | 8 | 0 | 3 | PAST | 1 | Yes | iBT + EBRT |
| gm074_0 | cancer | GEM1074 | T0 | 65 | E | C | 24.0 | 5 | 0 | 1 | NEVER | 1 | No | Surgery + Chemo |
| gm074_1 | cancer | GEM1074 | T1 | 65 | E | C | 24.0 | 6 | 22 | 1 | NA | NA | Yes | iBT |
| gm075_0 | healthy | GEM1075 | T0 | 57 | NA | AA | 27.8 | 6 | 0 | NA | NEVER | 1 | Yes | None |
| gm076_0 | healthy | GEM1076 | T0 | 62 | NA | AA | 24.8 | 5 | 0 | NA | PAST | 2 | No | None |
| gm077_0 | healthy | GEM1077 | T0 | 51 | NA | AA | 21.0 | 5 | 0 | NA | NEVER | 1 | Yes | None |
| gm078_0 | healthy | GEM1078 | T0 | 64 | NA | AA | 25.4 | 5 | 0 | NA | NEVER | 1 | No | None |
| gm080_0 | healthy | GEM1080 | T0 | 48 | NA | AA | 26.5 | 6.5 | 0 | NA | NEVER | 2 | Yes | None |
| gm081_0 | cancer | GEM1081 | T0 | 57 | E | AA | 32.0 | 6 | 0 | 1 | NEVER | 1 | Yes | Surgery |
| gm081_2 | cancer | GEM1081 | T1 | 57 | E | AA | 32.0 | 7 | 0 | 1 | NA | NA | No | iBT |
| gm082_0 | cancer | GEM1082 | T0 | 72 | E | AA | 37.6 | 6 | 0 | 1 | PAST | NA | Yes | Surgery |
| gm082_1 | cancer | GEM1082 | T1 | 72 | E | AA | 37.6 | NA | 22 | 1 | NA | NA | No | iBT |
| gm084_0 | healthy | GEM1084 | T0 | 54 | NA | A | 24.9 | 5 | 0 | NA | NEVER | 1 | NA | None |
| gm085_0 | healthy | GEM1085 | T0 | 56 | NA | AA | 41.2 | 5 | 0 | NA | NEVER | 1 | No | None |
| gm086_0 | cancer | GEM1086 | T0 | 60 | E | AA | 33.1 | 6 | 0 | 2 | NEVER | 2 | Yes | Surgery + Chemo |
| gm086_1 | cancer | GEM1086 | T1 | 60 | E | AA | 33.1 | 7 | 50.4 | 2 | NA | NA | Yes | EBRT |
| gm087_0 | healthy | GEM1087 | T0 | 52 | NA | AA | 53.6 | 6 | 0 | NA | NEVER | 1 | Yes | None |
| gm088_0 | cancer | GEM1088 | T0 | 55 | C | AA | 28.4 | 8 | 0 | 3 | NA | NA | NA | None |
| gm089_0 | healthy | GEM1089 | T0 | 53 | NA | C | 21.9 | NA | 0 | NA | NEVER | 3 | Yes | None |
| gm090_0 | healthy | GEM1090 | T0 | 53 | NA | AA | 27.6 | 5 | 0 | NA | NEVER | 1 | Yes | None |
| gm091_0 | cancer | GEM1091 | T0 | 52 | C | AA | 33.8 | 5 | 0 | 1 | PAST | 1 | No | Chemo |
| gm091_1 | cancer | GEM1091 | T1 | 52 | C | AA | 33.8 | 7 | 128 | 1 | NA | NA | Yes | iBT + EBRT |
| gm092_0 | healthy | GEM1092 | T0 | 45 | NA | AA | 28.3 | 7 | 0 | NA | NEVER | 1 | Yes | None |
| gm093_0 | cancer | GEM1093 | T0 | 62 | C | C | 22.6 | 6 | 0 | 1 | NA | NA | No | None |
| gm093_1 | cancer | GEM1093 | T1 | 62 | C | C | 22.6 | 5 | 127 | 1 | NA | NA | No | EBRT |
| gm094_0 | healthy | GEM1094 | T0 | 46 | NA | C | 26.6 | 7 | 0 | NA | PAST | 2 | Yes | None |
| gm095_0 | healthy | GEM1095 | T0 | 41 | NA | C | 24.7 | 7 | 0 | NA | NEVER | 1 | Yes | None |
| gm096_0 | cancer | GEM1096 | T0 | 66 | E | AA | 27.5 | 5 | 0 | 2 | NEVER | NA | No | Surgery |
| gm097_0 | cancer | GEM1097 | T0 | 48 | C | C | 20.5 | 6 | 0 | 2 | NEVER | 1 | No | None |
| gm098_0 | healthy | GEM1098 | T0 | 57 | NA | AA | 39.0 | 5 | 0 | NA | NEVER | NA | No | None |
| gm099_0 | cancer | GEM1099 | T0 | 46 | C | C | 30.3 | 5 | 0 | 2 | NA | NA | NA | None |
| gm100_0 | cancer | GEM1100 | T0 | 63 | E | C | 36.8 | 6 | 0 | 3 | PAST | 1 | Yes | Surgery |
| gm101_0 | healthy | GEM1101 | T0 | 49 | NA | C | 24.8 | 6 | 0 | NA | NEVER | 1 | Yes | None |
| gm103_0 | healthy | GEM1103 | T0 | 53 | NA | C | 23.4 | 6 | 0 | NA | NEVER | 2 | Yes | None |
| gm104_0 | healthy | GEM1104 | T0 | 50 | NA | C | 35.7 | 5 | 0 | NA | NEVER | 1 | No | None |
| gm106_0 | cancer | GEM1106 | T0 | 47 | E | AA | 39.7 | 4 | 0 | 1 | PAST | 1 | Yes | Surgery |
| gm106_1 | cancer | GEM1106 | T1 | 47 | E | AA | 39.7 | 6 | 25 | 1 | NA | NA | Yes | iBT |
| gm107_0 | healthy | GEM1107 | T0 | 54 | NA | AA | 42.0 | 4 | 0 | NA | NEVER | 1 | Yes | None |
| gm108_0 | cancer | GEM1108 | T0 | 45 | C | AA | 29.5 | 7 | 0 | 1 | NEVER | 1 | No | None |
| gm109_0 | cancer | GEM1109 | T0 | 64 | E | C | 49.1 | 7 | 0 | 2 | NEVER | 1 | Yes | None |
| gm110_0 | healthy | GEM1110 | T0 | 52 | NA | AA | 40.4 | 5 | 0 | NA | NEVER | 2 | Yes | None |
| gm111_0 | healthy | GEM1111 | T0 | 49 | NA | C | 21.5 | 3 | 0 | NA | NEVER | 1 | Yes | None |
| gm112_0 | healthy | GEM1112 | T0 | 52 | NA | AA | 28.3 | 5 | 0 | NA | NEVER | 1 | Yes | None |
| gm116_0 | cancer | GEM1116 | T0 | 74 | E | C | 35.9 | 7 | 0 | 1 | NEVER | 1 | Yes | Surgery |
| gm117_0 | cancer | GEM1117 | T0 | 69 | C | AA | 31.8 | 6 | 0 | 2 | NEVER | 1 | NA | None |
| gm121_0 | cancer | GEM1121 | T0 | 72 | E | AA | 46.4 | 6 | 0 | 3 | NA | NA | NA | Surgery |
| gm122_0 | cancer | GEM1122 | T0 | 57 | C | AA | 28.3 | 5 | 0 | 1 | CURRENT | NA | Yes | None |
| gm123_0 | cancer | GEM1123 | T0 | 47 | C | C | 19.3 | 6 | 0 | 4 | NEVER | 1 | No | None |
| gm124_0 | cancer | GEM1124 | T0 | 52 | E | C | 40.4 | 6 | 0 | 1 | NEVER | 1 | Yes | Surgery |
| gm126_0 | cancer | GEM1126 | T0 | 56 | E | A | 27.0 | 6 | 0 | 1 | NEVER | 1 | Yes | Surgery |
| gm127_0 | cancer | GEM1127 | T0 | 70 | C | C | 31.1 | 6 | 0 | 3 | NEVER | 3 | No | Surgery |
| gm128_0 | cancer | GEM1128 | T0 | 63 | E | C | 20.8 | 5.5 | 0 | NA | NEVER | 1 | Yes | Surgery |
| gm129_0 | cancer | GEM1129 | T0 | 79 | E | A | 30.1 | 6 | 0 | 1 | NEVER | 1 | No | Surgery |
| gm130_0 | cancer | GEM1130 | T0 | 59 | C | AA | 23.0 | 8 | 0 | 3 | NEVER | 1 | No | None |
| gm131_0 | cancer | GEM1131 | T0 | 80 | E | C | 28.3 | 6 | 0 | 1 | PAST | 1 | Yes | Surgery + Chemo |
| gm132_0 | cancer | GEM1132 | T0 | 66 | E | AA | 32.4 | 6 | 0 | 1 | NEVER | NA | NA | Surgery |
| gm133_0 | cancer | GEM1133 | T0 | 30 | C | AA | 19.9 | 6 | 0 | NA | NEVER | 1 | No | None |
| gm134_0 | cancer | GEM1134 | T0 | 82 | E | AA | 31.8 | 7 | 0 | NA | PAST | 1 | No | Surgery |
| gm135_0 | cancer | GEM1135 | T0 | 71 | E | AA | 30.8 | 7 | 0 | 1 | NEVER | 1 | Yes | Surgery |
| gm137_0 | cancer | GEM1137 | T0 | 51 | C | C | 25.7 | NA | 0 | 1 | NEVER | 1 | Yes | Surgery |
| yn006_0 | cancer | GYN6006 | T0 | 55 | E | AA | 28.5 | NA | 0 | 3 | NA | 1 | Yes | Surgery + Chemo |
| yn006_2 | cancer | GYN6006 | T1 | 55 | E | AA | 28.5 | NA | 0 | 3 | NA | NA | Yes | iBT |
| yn008_0 | cancer | GYN6008 | T0 | 63 | E | AA | 44.2 | NA | 0 | 2 | NA | 1 | Yes | Surgery |
| yn008_1 | cancer | GYN6008 | T1 | 63 | E | AA | 44.2 | NA | NA | 2 | NA | NA | Yes | iBT |
| yn009_0 | cancer | GYN6009 | T0 | 63 | E | AA | 42.6 | NA | 0 | 1 | NA | NA | No | Surgery + Chemo |
| yn010_0 | cancer | GYN6010 | T0 | 56 | E | C | 31.0 | NA | 0 | 1 | NA | NA | Yes | Surgery |
| yn010_2 | cancer | GYN6010 | T1 | 56 | E | C | 31.0 | NA | 0 | 1 | NA | NA | Yes | iBT |
| yn011_0 | cancer | GYN6011 | T0 | 49 | C | AA | 20.8 | NA | 0 | 3 | NA | NA | No | Surgery + Chemo |
| yn012_0 | cancer | GYN6012 | T0 | 59 | C | AA | 32.9 | NA | 0 | 3 | NA | NA | Yes | Chemo |
| yn013_0 | cancer | GYN6013 | T0 | 42 | C | AA | 24.1 | NA | 0 | 1 | NA | NA | Yes | Surgery + Chemo |
| yn013_1 | cancer | GYN6013 | T1 | 42 | C | AA | 24.1 | NA | NA | 1 | NA | NA | Yes | iBT |
| yn015_0 | cancer | GYN6015 | T0 | 35 | C | C | 43.2 | NA | 0 | 1 | NA | NA | Yes | Surgery + Chemo |
| yn015_1 | cancer | GYN6015 | T1 | 35 | C | C | 43.2 | NA | NA | 1 | NA | NA | No | iBT |
| yn017_0 | cancer | GYN6017 | T0 | 42 | C | C | 40.2 | NA | 0 | 2 | NA | NA | Yes | Surgery + Chemo |
| yn022_0 | cancer | GYN6022 | T0 | 44 | C | AA | 30.6 | NA | 0 | 2 | NA | NA | No | Surgery + Chemo |
| yn022_1 | cancer | GYN6022 | T1 | 44 | C | AA | 31.6 | NA | NA | 2 | NA | NA | Yes | iBT |
| yn023_0 | cancer | GYN6023 | T0 | 32 | C | C | 26.1 | NA | 0 | 3 | NA | NA | Yes | Surgery + Chemo |
| yn023_1 | cancer | GYN6023 | T1 | 32 | C | C | 26.1 | NA | NA | 3 | NA | NA | Yes | iBT |
| yn024_0 | cancer | GYN6024 | T0 | 63 | E | AA | 31.8 | NA | 0 | 2 | NA | NA | Yes | Surgery |
| yn025_0 | cancer | GYN6025 | T0 | 70 | E | AA | 27.7 | NA | 0 | NA | NA | NA | No | Surgery + Chemo |
| yn028_0 | cancer | GYN6028 | T0 | 74 | E | AA | 27.6 | NA | 0 | 2 | NA | NA | Yes | Surgery + Chemo |

**CANCER TYPE** Cancer type from medical record C: Cervical, E: Endometrial

**ANCESTRY** Self reported ancestry AA; African American, C: Caucasian, A: Asian

**ALCOHOL** What is your alcohol consumption in the past ONE year? 1, < 1 drink/week | 2, 1-4 drinks/w | 3, 5-14 drinks/w | 4, 15-29 drinks/w| 5, >= 30 drinks/w

**SMOKER** Have you smoked at least 100 cigarettes, cigars, or e-cigarettes in your ENTIRE LIFE? NEVER, PAST, CURRENT

**Sexual_Intercourse_4W** Have you had sexual intercourse during the past 4 weeks? Yes, No

**HRT-EVER** Have you ever used Hormone Replacement Therapy (HRT)? Yes, No

**Treatment** Type of cancer treatment redtrived from the medical chart record Surgery, Chemotherapy, EBRT, iBT

| **Part B: Sequence Dataset Quality** | | | |
| --- | --- | --- | --- |
| **SampleID** | **Raw Reads** | **Denoised +QC** | **Merged** |
| gm001_2 | 31834 | 20057 | 19512 |
| gm002_0 | 38969 | 28500 | 28206 |
| gm003_0 | 34069 | 24522 | 24343 |
| gm004_0 | 35116 | 24339 | 24152 |
| gm006_0 | 42778 | 29277 | 28996 |
| gm007_0 | 45079 | 32073 | 31944 |
| gm008_0 | 37197 | 27055 | 27021 |
| gm009_0 | 18232 | 12559 | 12352 |
| gm010_0 | 50252 | 10017 | 9430 |
| gm011_0 | 46073 | 32293 | 31837 |
| gm013_0 | 72888 | 52217 | 52023 |
| gm014_0 | 45334 | 31886 | 31786 |
| gm015_0 | 34113 | 23986 | 22801 |
| gm016_0 | 25156 | 18372 | 18365 |
| gm017_1 | 70201 | 49187 | 48853 |
| gm018_0 | 47497 | 33353 | 32835 |
| gm018_1 | 59838 | 41973 | 41694 |
| gm019_0 | 26322 | 19000 | 18954 |
| gm020_0 | 41402 | 29967 | 29860 |
| gm021_0 | 46543 | 32183 | 31962 |
| gm022_0 | 29340 | 20195 | 19982 |
| gm022_1 | 38644 | 26010 | 25624 |
| gm024_0 | 35412 | 25155 | 24732 |
| gm024_1 | 35960 | 26072 | 25916 |
| gm025_0 | 19737 | 13928 | 13765 |
| gm027_0 | 26401 | 19086 | 19046 |
| gm027_1 | 16224 | 10511 | 10184 |
| gm028_0 | 18096 | 8748 | 8445 |
| gm029_0 | 30651 | 21654 | 21403 |
| gm030_0 | 63487 | 43909 | 43583 |
| gm031_0 | 19477 | 10754 | 10507 |
| gm032_0 | 18318 | 8437 | 8180 |
| gm033_0 | 43484 | 30996 | 30793 |
| gm035_0 | 57186 | 37611 | 37328 |
| gm036_0 | 35646 | 24922 | 24705 |
| gm037_0 | 29721 | 21281 | 21246 |
| gm037_1 | 27061 | 19313 | 19088 |
| gm038_0 | 40915 | 29601 | 29595 |
| gm039_0 | 40689 | 28770 | 28182 |
| gm040_0 | 42505 | 29198 | 28807 |
| gm041_0 | 20022 | 14347 | 14168 |
| gm042_0 | 24085 | 17315 | 17100 |
| gm043_0 | 33621 | 24138 | 24022 |
| gm044_0 | 39068 | 24902 | 24590 |
| gm044_1 | 42079 | 29248 | 29034 |
| gm045_0 | 29022 | 20693 | 20660 |
| gm046_0 | 32739 | 8826 | 8006 |
| gm047_0 | 22927 | 16079 | 15999 |
| gm049_0 | 29627 | 20536 | 20264 |
| gm050_0 | 31588 | 22282 | 21717 |
| gm051_0 | 32511 | 22880 | 22823 |
| gm052_0 | 33594 | 23116 | 23017 |
| gm053_0 | 24425 | 16939 | 16520 |
| gm054_0 | 20599 | 14482 | 14276 |
| gm054_1 | 19733 | 13536 | 13020 |
| gm055_0 | 19563 | 13475 | 13263 |
| gm056_0 | 26827 | 19404 | 19322 |
| gm056_1 | 30503 | 21995 | 21944 |
| gm057_0 | 29314 | 20466 | 20210 |
| gm058_0 | 36888 | 26217 | 26049 |
| gm059_0 | 30200 | 22035 | 21861 |
| gm060_0 | 13685 | 4871 | 4390 |
| gm061_0 | 15024 | 9151 | 8533 |
| gm117_0 | 35573 | 25426 | 25305 |
| gm062_0 | 36178 | 26459 | 26329 |
| gm063_0 | 39449 | 28516 | 28459 |
| gm064_0 | 48018 | 33294 | 32999 |
| gm065_0 | 28221 | 19422 | 19145 |
| gm065_1 | 38949 | 12694 | 12274 |
| gm066_0 | 34616 | 23778 | 23620 |
| gm067_0 | 23593 | 17157 | 17017 |
| gm068_0 | 41378 | 29264 | 28908 |
| gm069_0 | 55092 | 38899 | 38473 |
| gm070_0 | 372768 | 263584 | 262095 |
| gm071_0 | 25975 | 18302 | 18165 |
| gm071_1 | 39261 | 24357 | 24145 |
| gm072_0 | 23686 | 16531 | 16312 |
| gm073_0 | 23728 | 15207 | 14853 |
| gm073_1 | 59228 | 40177 | 39972 |
| gm074_0 | 33149 | 19950 | 19649 |
| gm074_1 | 5104 | 2431 | 2308 |
| gm075_0 | 37883 | 20143 | 19523 |
| gm076_0 | 47705 | 32678 | 32086 |
| gm077_0 | 92734 | 65558 | 65208 |
| gm078_0 | 47040 | 26186 | 25485 |
| gm080_0 | 43082 | 25819 | 25455 |
| gm081_0 | 45663 | 26324 | 25857 |
| gm081_2 | 39819 | 20240 | 19719 |
| gm082_0 | 51034 | 36342 | 36059 |
| gm082_1 | 54018 | 37530 | 36880 |
| gm084_0 | 40371 | 29313 | 29276 |
| gm085_0 | 21401 | 15224 | 15156 |
| gm086_0 | 27664 | 19574 | 19273 |
| gm086_1 | 41442 | 29615 | 29495 |
| gm087_0 | 49860 | 34673 | 34188 |
| gm088_0 | 52928 | 37904 | 37661 |
| gm089_0 | 39515 | 28000 | 27791 |
| gm090_0 | 43691 | 31647 | 31571 |
| gm091_0 | 80761 | 57248 | 56637 |
| gm091_1 | 39160 | 26664 | 26304 |
| gm092_0 | 37957 | 27012 | 26561 |
| gm093_0 | 23709 | 16860 | 16769 |
| gm093_1 | 45719 | 28634 | 28435 |
| gm094_0 | 50125 | 35420 | 35331 |
| gm095_0 | 27321 | 18856 | 18819 |
| gm096_0 | 42112 | 27445 | 26998 |
| gm097_0 | 29625 | 20310 | 20185 |
| gm098_0 | 27066 | 19597 | 19477 |
| gm099_0 | 27433 | 19008 | 18864 |
| gm100_0 | 22329 | 14969 | 14696 |
| gm101_0 | 41841 | 30075 | 29947 |
| gm103_0 | 21471 | 7109 | 6579 |
| gm104_0 | 49482 | 34872 | 34437 |
| gm106_0 | 61606 | 44923 | 44507 |
| gm106_1 | 35668 | 25775 | 25541 |
| gm107_0 | 48513 | 34602 | 34292 |
| gm108_0 | 27772 | 19571 | 19301 |
| gm109_0 | 28539 | 19956 | 19736 |
| gm110_0 | 48513 | 33971 | 33530 |
| gm111_0 | 35411 | 25381 | 25229 |
| gm112_0 | 47418 | 32704 | 32550 |
| gm116_0 | 29792 | 20901 | 20716 |
| gm117_0 | 63654 | 41170 | 40457 |
| gm121_0 | 40637 | 28515 | 28421 |
| gm122_0 | 21393 | 14826 | 14584 |
| gm123_0 | 47998 | 34307 | 34028 |
| gm124_0 | 55652 | 40196 | 39944 |
| gm126_0 | 63018 | 44511 | 44424 |
| gm127_0 | 22326 | 16105 | 16031 |
| gm128_0 | 22344 | 15288 | 15155 |
| gm129_0 | 31281 | 22052 | 21854 |
| gm130_0 | 23297 | 13792 | 13457 |
| gm131_0 | 45857 | 32191 | 32053 |
| gm132_0 | 47141 | 32587 | 32384 |
| gm133_0 | 59789 | 42973 | 42412 |
| gm134_0 | 33056 | 23449 | 23223 |
| gm135_0 | 43014 | 30158 | 29910 |
| gm137_0 | 32404 | 23513 | 23250 |
| yn006_0 | 22777 | 15754 | 15677 |
| yn006_2 | 15744 | 8567 | 8345 |
| yn008_0 | 39509 | 28785 | 28722 |
| yn008_1 | 37123 | 27092 | 26891 |
| yn009_0 | 19076 | 13550 | 13343 |
| yn010_0 | 31681 | 22204 | 22108 |
| yn010_2 | 18852 | 13117 | 13023 |
| yn011_0 | 47208 | 33088 | 33046 |
| yn012_0 | 18660 | 13232 | 13079 |
| yn013_0 | 32837 | 23480 | 23062 |
| yn013_1 | 34183 | 23880 | 23679 |
| yn015_0 | 47480 | 33870 | 33656 |
| yn015_1 | 35530 | 25835 | 25542 |
| yn017_0 | 28319 | 19962 | 19767 |
| yn022_0 | 18475 | 12906 | 12631 |
| yn022_1 | 25601 | 18430 | 18231 |
| yn023_0 | 48640 | 34530 | 34197 |
| yn023_1 | 33914 | 23869 | 23801 |
| yn024_0 | 38070 | 27312 | 27181 |
| yn025_0 | 53236 | 7938 | 7502 |
| yn028_0 | 42696 | 29455 | 29109 |
