# Supplementary material for: Comparison of vaginal microbiota in gynecologic cancer patients pre‐ and post‐radiation therapy and healthy women
Source: Cancer Med. 2020 Apr 1;9(11):3714–24. doi: 10.1002/cam4.3027 (PMC7286461; doi:10.1002/cam4.3027)
Supplement: Supplementary file 3 — Table S2 [file CAM4-9-3714-s003.docx]

**Supportive Table 2**: Inclusion and exclusion criteria of recruited subjects used in this study

| **Inclusion Criteria:** |
| --- |
| ☐ Post-menopausal |
| ☐ Previous HPV or other STD is **not exclusionary if treated and cleared** |
| ☐ **Well-controlled** Type 2 diabetes is acceptable ☐ Hypothyroidism **is not** exclusionary if controlled (i.e. treated with medication) |
| ☐ Must be able to read, write, and speak English |
| ☐ Diagnosed with either **Cervical** or **Endometrial** cancer [For Cancer cohort] |
| ☐ Scheduled for Radiation Therapy (prior surgery and/or chemotherapy is permitted) [For Cancer cohort] |
| **Cohort Exclusion Criteria:** |
| ☐ History of metastatic or other primary cancer or previous radiation therapy |
| ☐ Co-morbidities including: HIV, cystic fibrosis, poorly controlled diabetes, autoimmune disease, STDs, fungal infection, Crohn’s Disease, Ulcerative colitis, Hepatitis C, HSV (Herpes Simplex) |
| ☐ Exclude diabetic patients with neuropathy, history of gout, high BMI (>49), and/or taking Neurontin (gabapentin)—**poorly controlled** ☐ Type 1 Diabetes |
| ☐ Use of HRT (hormone replacement therapy) and/or vaginal estrogens within 4 weeks of baseline |
| ☐ Use of antibiotics, corticosteroids, or topical estrogen within 4 weeks requires rescheduling or exclusion |
| ☐Use of interferon(s) ***or*** immunosuppressive therapies or *megestrol* Ex. interferon beta-1a (i.e. Rebif, Plegridy, etc.) |
